# S234. ONE-YEAR OUTCOME AND USE OF CLOZAPINE IN FIRST-EPISODE SCHIZOPHRENIA

**DOI:** 10.1093/schbul/sby018.1021

**Published:** 2018-04-01

**Authors:** Petros Drosos, Kolbjørn Brønnick, Rune Kroken, Inge Joa, Jan Olav Johannessen, Tor Ketil Larsen

**Affiliations:** 1 Stavanger University Hospital; 2 TIPS – Centre for Clinical Research in Psychosis, Stavanger University Hospital; 3 Haukeland University Hospital, University of Bergen; 4 University of Bergen

## Abstract

**Background:**

The aim of this study is to examine the one-year outcome in a cohort of patients with a first-episode core schizophrenia diagnosis (schizophrenia, schizophreniform psychosis, schizoaffective disorder) and the use of clozapine in the non-remitted patients at one-year control.

**Methods:**

The population studied is the patients who were included with a first-episode psychosis in the TIPS project in the period 01.01.2002-31.12.2010 and had a core schizophrenia diagnosis. We divided the patients into two groups according to their remission status at one-year follow up and compared their main characteristics. We then performed a digital search in the hospitaĹs journal of the non-remitted group for the words “clozapine” and “Leponex”.

**Results:**

Out of the 78 patients with first-episode core schizophrenia diagnosis included in the TIPS project during the examined period, 53 were continuously psychotic at one-year follow up. The one-year remission rate for our sample was therefore 32%. All of the non-remitted patients during the first year could be eligible for clozapine, but clozapine was considered to only 3 of them (5.7 %) and only two of them were offered clozapine. The mean number of periods with antipsychotic treatment in this group was four (4).

**Discussion:**

The findings in our study show firstly a surprisingly low one-year remission rate for first-episode schizophrenia (32 %). This is much lower than what corresponding studies of the last years show. Our results also prove the underutilization of clozapine in non-remitted patients with a first-episode core schizophrenia diagnosis. Therefore, the clinicians did not follow the recommended guidelines for the treatment of schizophrenia. The possible reasons for this low use of clozapine will be discussed, but it was not possible to verify them as there was not found any relevant information in the patients’ files.

